# Effects of *RET, NRG1* and *NRG3* Polymorphisms in a Chinese Population with Hirschsprung Disease

**DOI:** 10.1038/srep43222

**Published:** 2017-03-03

**Authors:** Dehua Yang, Jun Yang, Shuai Li, Meng Jiang, Guoqing Cao, Li Yang, Xi Zhang, Ying Zhou, Kang Li, Shao-tao Tang

**Affiliations:** 1 Department of Pediatric Surgery, Union Hospital, Tongji Medical College, Huazhong University of Science and Technology, Wuhan 430022, China; 2 Department of Pediatric Surgery, Wuhan Children’s Hospital (Wuhan Maternal and Child Healthcare Hospital), Tongji Medical College, Huazhong University of Science and Technology, Wuhan 430015, China

## Abstract

The *RET* proto-oncogene was identified as a major locus involved in Hirschsprung disease (HSCR). A genome-wide association study (GWAS) and whole exome sequencing identified *NRG1* and *NRG3* as additional HSCR susceptibility loci. We investigated the effects of *RET* (rs2506030 and rs2435357), *NRG1* (rs2439302, rs16879552 and rs7835688) and *NRG3* (rs10748842, rs10883866 and rs6584400) polymorphisms in a Chinese population with HSCR. We assessed single nucleotide polymorphisms (SNPs) in the *RET, NRG1* and *NRG3* genes in a cohort of 362 sporadic HSCR patients and 1,448 normal controls using a TaqMan genotyping assay. Significant associations were found between HSCR risk and rs2506030, rs2435357, rs2439302 and rs7835688 (odds ratio [OR] 1.64, *P* = 1.72E-06; 2.97, *P* = 5.15E-33; 1.84, *P* = 9.36E-11; and 1.93, *P* = 1.88E-12, respectively). Two locus analyses of SNPs indicated increased disease risks of HSCR between NRG1 rs2439302 and RET rs2435357 or rs2506030. *RET* rs2506030 (GG genotype) and rs2435357 (TT genotype), in combination with *NRG1* rs2439302 (GG genotype), were strongly associated with the highest risk of HSCR (OR = 56.53, *P* = 4.50E-07) compared with the two loci or a single SNP of either *RET* or *NRG1*. Our results support the association between genetic variation of *RET* and *NRG1* and susceptibility to HSCR in the Chinese population.

Hirschsprung disease (HSCR, OMIM 142623) or aganglionic megacolon is one of the most common developmental disorders of the enteric nervous system (ENS) in children. It is characterized by the absence of intramural ganglion cells in the myenteric and submucosal plexuses of the gastrointestinal tract, resulting in tonic contraction of the affected segment, massive distension of the proximal bowel and intestinal obstruction^1^,^2^,^3^. This common congenital disorder, which occurs in 1:5,000 live births, shows a significant difference among ethnic groups (10, 15, 21 and 28 per 100,000 live births among Hispanics, Caucasian-Americans, African-Americans, and Asians, respectively)^4^. Based on the location of aganglionosis, the patients can be classified into three anatomical types: short-segment HSCR (S-HSCR; accounting for 80% of cases), long-segment HSCR (L-HSCR; accounting for 15–20% of cases) and total colonic aganglionosis (TCA; accounting for less than 5% of cases)^5^. HSCR also shows sex-dependent penetrance^6^ and appears to be a non-Mendelian malformation with low penetrance^7^.

The genetic reasons for the variable phenotypes, including the segment length of aganglionosis, sex bias with a male preponderance, high recurrence among descendants and associated phenotypes, remain largely unknown^8^. Through linkage mapping and mouse model analysis, a series of susceptible genes were found to contribute to the development of the ENS, including *RET* (OMIM 164761), *EDNRB* (OMIM 131244), *GDNF* (OMIM 600837), *ECE1* (OMIM 164761), *NRTN* (OMIM 602018), *PHOX2B* (OMIM 603851), *SOX10* (OMIM 602229), *EDN3* (OMIM 131242), *ZFHX1B* (OMIM 605802), *L1CAM* (OMIM 308840), *TCF4* (OMIM 602272), and *KBP10* (OMIM 609367)^6^,^9^,^10^,^11^,^12^,^13^,^14^,^15^,^16^,^17^,^18^,^19^. These genes encode transcription factors, receptors, ligands, and other cellular elements involved in the development of ENS^20^. During the past several years, studies have revealed molecular anomalies or novel genes associated with HSCR, such as copy number variation^5^,^21^, polymorphisms in the 3′ untranslated region^22^, and gene-gene interactions^23^, as well as microRNA interactions^24^. However, these genes cannot account for all of the HSCR cases. Thus, there might be other chromosomal abnormalities responsible for HSCR.

The Neuregulin 1 (*NRG1*) and Neuregulin 3 (*NRG3*) genes, located on chromosomes 8p12 and 10q22-q23, respectively, are members of the neuregulin family. They regulate glial commitment, survival, proliferation, and differentiation. Neuregulins belong to the epithelial growth factor family that induces growth and differentiation of epithelial, muscle, and glial cells in culture^25^. *NRG1* is a signaling protein that mediates cell-cell interactions and plays an important role in the development of the nervous system, breast, heart, and other organs. It has also been related to schizophrenia and HSCR in some genome-wide association studies (GWAS) in individuals of Asian descent^26^,^27^ as well as survival of enteric neural crest cells (ENCCs)^28^. The *NRG1/ErbB* complex promotes postnatal maintenance of the ENS and neuronal survival through other biological functions^10^,^29^. As a paralog of the *NRG1* analogue, *NRG3* shares the same receptor, *ErbB4*, with *NRG1*, and both genes may play important roles in the development of the embryonic cerebral cortex through regulation of cortical cell differentiation, proliferation, and migration^30^. Our previous study confirmed exome genetic variation in *NRG3* as a risk factor for HSCR^9^. The *RET* gene, which encodes a tyrosine-kinase receptor, is the most compelling susceptible gene associated with HSCR, and its expression is crucial for the development of the ENS. Mutations in the *RET* gene account for approximately 50% of familial cases and 15–20% of sporadic HSCR cases^31^.

Some previous studies reported that rs7835688 and rs16879552 at *NRG1* were associated with susceptibility to HSCR^26^,^32^. It has been reported that a thyroid cancer variant (rs2439302) and one of the two originally reported variants (rs7835688) associated with HSCR were positively correlated on the basis of HapMap data from Asian individuals. Hence, the same sequence variant in *NRG1* likely affects circulating thyroid-stimulating hormone levels and the risk of HSCR and thyroid cancer^33^. Our previous exome sequencing identified coding region polymorphisms at *NRG3* and indicated that this novel gene is associated with susceptibility to HSCR in a Chinese population. However, we did not sequence the intron regions. A fine-mapping study identified the genetic variants rs10883866, rs10748842 and rs6584400 in intron 1 of *NRG3* as risk factors that are associated with the development of the nervous system and that influence *NRG3* gene expression^30^,^34^. However, until recently, we did not know whether these three variants in *NRG3* were related to the risk of HSCR. *RET* variations appear to have a genetic association with HSCR. In a study conducted by Li, rs2435357 and rs2506030 in *RET* were confirmed to be risk factors for HSCR in a Chinese population. Among these variants, rs2435357 was investigated in different ethnicities^35^,^36^,^37^. However, there have fewer subsequent studies on rs2506030 and HSCR^37^,^38^. Therefore, we wanted to replicate this study and determine the strength of the associations between these two variants in *RET* and susceptibility to HSCR in our samples.

Accordingly, we designed this case-controlled study to investigate the relevance of these eight SNPs to the pathogenesis of HSCR, and we explored the SNP-SNP interactions with an aim to evaluate the disease risk level. Moreover, we detected differential gene expression levels by qRT-PCR and western blotting in colon tissues from patients.

## Results

### Characteristics of study subjects

The characteristics of the 362 HSCR patients and 1,448 healthy subjects are summarized in Table 1. There were 275 (75.97%) male and 87 (24.03%) female HSCR subjects, while 1,100 (75.97%) male subjects and 348 (24.03%) female subjects were included in the healthy control group. The cases and controls were well matched regarding gender distribution (*P* = 1.00). All the patients were diagnosed with S-HSCR.

### Association analysis of candidate polymorphisms with HSCR risk

For the eight genetic markers at *RET* (rs2506030 and rs2435357), *NRG1* (rs2439302, rs7835688 and rs16879552), and *NRG3* (rs10748842, rs10883866 and rs6584400), a Hardy-Weinberg equilibrium test was performed on HSCR cases and controls. Genotype distributions were in Hardy-Weinberg equilibrium for the polymorphisms in both the HSCR cases and the controls (Chi-squared test, *P* > 0.05), and the statistical test power was >95.0%. The distribution of the eight SNPs in the HSCR cases and controls is shown in Table 2 and Supplementary Table S1. The SNPs rs2435357, rs2506030, rs2439302 and rs7835688 showed a positive correlation with HSCR susceptibility, with an OR greater than 1.0 (*P* < 0.05). The risk allele frequencies for these three SNPs were significantly higher in HSCR cases than in controls (rs2439302 risk allele G: 26.9% vs. 18.6%, *P* = 9.36E-11; rs7835688 risk allele C: 30.1% vs. 18.3%, *P* = 1.88E-12; rs2435357 risk allele T: 75.1% vs. 50.4%, *P* = 5.15E-33; rs2506030 risk allele G: 80.1% vs. 71.1%, *P* = 1.72E-06), with OR values of 1.84 (95% confidence interval [CI] = 1.53–2.21), 1.93 (95% CI = 1.60–2.32), 2.97 (95% CI = 2.47–3.57) and 1.64 (95% CI = 1.34–2.00), respectively.

Under a multivariate logistic regression model, individuals with CG and GG genotypes of rs2439302 had a significantly increased risk of HSCR with an OR of 1.49 (95% CI = 1.16–1.91, *P* = 2.00E-03) and 5.01 (95% CI = 3.14–8.00, *P* = 1.57E-11), respectively, compared to those with the CC genotype. Thus, the CG and GG genotype carriers had a 1.49- and 5.01-fold, respectively, elevated risk of developing HSCR compared with the CC genotype carriers. Moreover, the G allele was significantly associated with an increased risk of HSCR in the additive (OR = 1.85, 95% CI = 1.53–2.23, *P* = 2.15E-10) and allelic models (OR = 1.84, 95% CI = 1.53–2.21, *P* = 9.36E-11). Likewise, a significant association between this polymorphism and increased HSCR risk was found in the dominant (OR = 1.77, 95% CI = 1.40–2.24, *P* = 1.64E-06) and recessive models (OR = 4.32, 95% CI = 2.73–6.82, *P* = 3.70E-10). Similarly, compared to those with the GG genotype, individuals with the GC and CC genotypes of rs7835688 had an increased risk of HSCR (GC OR = 1.58, (95% CI = 1.22–2.04, *P* = 4.62E-04; CC OR = 3.59, 95% CI = 2.41–5.33, *P* = 2.66E-10). An increased risk of HSCR was found in the additive (OR = 1.78, 95% CI = 1.50–2.12, *P* = 9.71E-11), recessive (OR = 3.08, 95% CI = 2.10–4.53, *P* = 1.02E-08) and dominant models (OR = 1.89, 95% CI = 1.49–2.39, *P* = 1.09E-07). For the rs2435357 and rs2506030 loci, patients who carried the TT or GG genotype had a significantly increased HSCR risk compared to those who carried the CC or AA genotype, with OR values of 3.22 (95% CI = 1.79–5.79, *P* = 9.50E-05) and 7.46 (95% CI = 4.85–11.46, *P* = 4.74E-20), respectively. Moreover, significant correlations between rs2506030 or rs2435357 and HSCR risk were also identified in the additive, recessive and dominant models (rs2506030 OR: 1.60, 95% CI = 1.31–1.95, *P* = 4.47E-06; 1.64, 95% CI = 1.28–2.09, *P* = 7.13E-05; and 2.80, 95% CI = 1.57–5.00, *P* = 1.00E-03, respectively; rs2435357 OR: 3.25, 95% CI = 2.67–3.96, *P* = 1.32E-31; 4.63, 95% CI = 3.63–5.90, *P* = 2.35E-35; and 3.48, 95% CI = 2.31–5.25, *P* = 2.89E-09, respectively).

However, no genetic correlation was found between HSCR and the three SNPs at *NRG3* (rs10748842, rs10883866 and rs6584400) or the SNP at *NRG1* (rs16879552) when comparing the risk allele with the non-risk allele or in the additive, recessive and dominant models. In addition, no relevancy was observed in the heterozygote or the homozygote variant (see Supplementary Table S1).

### Correlations between *RET* and *NRG1* expression in HSCR ganglionic colon tissues and genetic variants of rs2435357, rs2506030 and rs2439302

**Figure 1:**
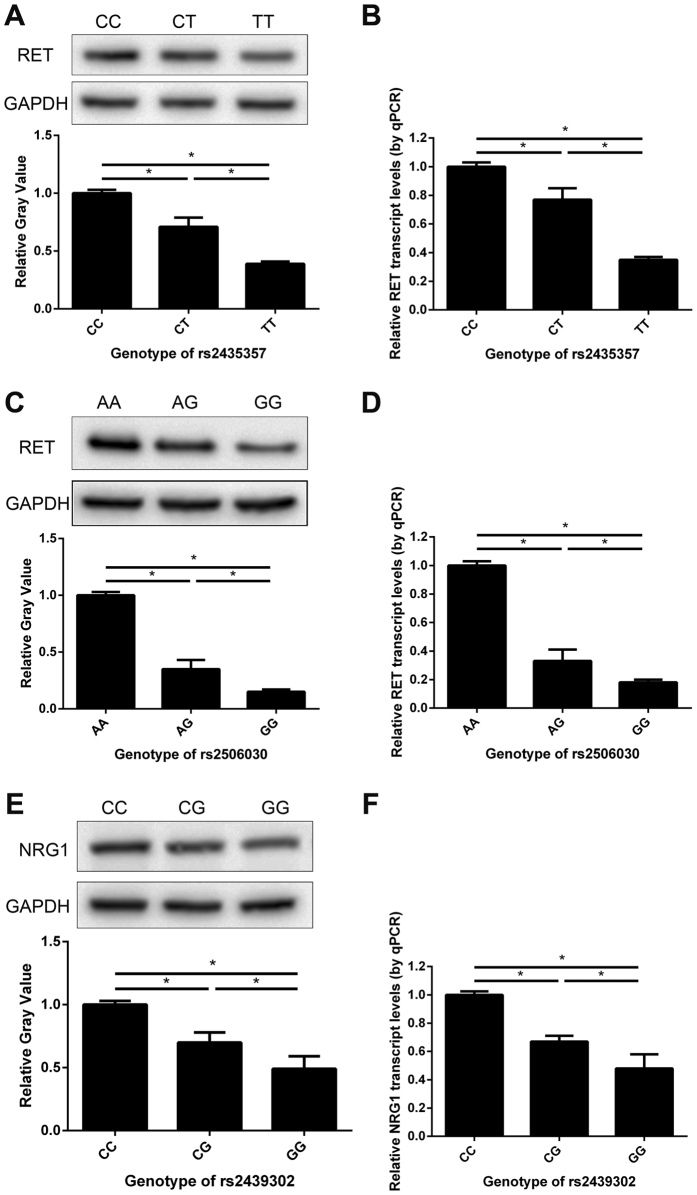
Correlations between *RET* and *NRG1* expression in HSCR ganglionic colon tissues and genetic variants of rs2435357, rs2506030 and rs2439302. (**A**,**C** and **E**), Western blot and respective gray-level analysis of *NRG1* and *RET* expression levels in HSCR patients of three genotypes (rs2435357: CC, CT and TT; rs2506030: AA, AG and GG; and rs2439302: CC, CG and GG). RT-qPCR analysis of *RET* (**B** and **D**) and *NRG1* (**F**) expression levels in HSCR patients of three genotypes (rs2435357: CC, CT and TT; rs2506030: AA, AG and GG; and rs2439302: CC, CG and GG). *P < 0.05. n = 10 in each group. The samples were derived from the same experiment, and the blots were processed in parallel. Full-size blots are presented in Supplementary Figure S3.

Because rs2435357 and rs2506030 are located on the same chromosome and were identified to be associated with HSCR, we calculated the linkage disequilibrium (LD) of these two SNPs. The r^2^ value of these two SNPs is 0.041, which suggest they are in low association and have independent genetic effects for HSCR. The two variants at the *NRG1* locus, rs7835688 and rs2439302, are in high LD (r^2^ = 0.95). This result indicated that rs7835688 and rs2439302 are strongly associated and in high allelic identity. In this way, one variant shows significant association with HSCR, the other variant is expected to also show disease association by the phenomenon of LD. Therefore, we report rs2439302 as representative of the association. To explore whether any of the three genotypes in rs2435357, rs2506030 and rs2439302 were associated with *RET* and *NRG1* gene expression, we performed western blotting and real-time quantitative reverse transcription PCR (RT-qPCR) on ganglionic colon tissues obtained from HSCR patients. Compared with rs2435357-CC or rs2506030-AA, relative *RET* protein expression was decreased in rs2435357-CT and rs2435357-TT or rs2506030-AG and rs2506030-GG (Fig. 1A and C). Image analysis of western blots demonstrated a significant decrease of *RET* protein expression (*P* < 0.05) (Fig. 1A and C). RT-qPCR revealed that *RET* was down-regulated in HSCR gut tissues. The relative expression of *RET* was lower in heterozygous and homozygous carriers of the HSCR risk allele than that in homozygous wild type subjects (*P* < 0.05) (Fig. 1B and D). For rs2439302, *NRG1* mRNA and protein expression was lowest in individuals homozygous for the G risk allele and highest in those homozygous for the CC genotype (*P* < 0.05; Fig. 1E and F).

### Joint effects of rs2439302 (rs7835688) at *NRG1* and rs2506030 and/or rs2435357 at *RET* on HSCR risk

From the above results, we identified rs2439302, rs7835688, rs2506030 and rs2435357 as the most relevant markers associated with HSCR in a Chinese population. As shown in Table 3, we further analyzed the combination of these two genes and found a significantly increased HSCR risk with an increasing number of risk alleles. Compared with other genotypes, patients carrying the homozygous risk alleles of rs2439302-GG and rs2506030-GG or rs2439302-GG and rs2435357-TT had the highest HSCR risk, with an OR of 25.69 (95% CI = 10.11–65.29, *P* = 9.12E-12) and 25.57 (95% CI = 10.88–60.07, *P* = 1.02E-13), respectively. Simultaneously, these three SNPs that were associated with HSCR risk were analyzed for cumulative effects. The patients carrying homozygous risk alleles also had the highest HSCR risk with an OR of 56.53 (95% CI = 12.16–262.83, *P* = 4.50E-07; Table 4). Furthermore, we obtained similar results for patients harboring two (rs7835688-CC and rs2506030-GG or rs7835688-CC and rs2435357-TT) or three (rs7835688-CC, rs2506030-GG and rs2435357-TT) homozygous risk alleles, with an OR of 24.13 (95% CI = 9.54–61.05, *P* = 1.80E-11), 24.10 (95% CI = 9.82–59.13, *P* = 3.68E-12), 54.83 (95% CI = 10.94–274.74, *P* = 1.12E-06), respectively (Supplementary Table S2).

## Discussion

HSCR is a multifactorial genetic disorder with high heritability (>80%, depending on the sex of the proband and affected sibling), high sibling recurrence risk (200-fold greater than the population), significant gender bias, and complex inheritance patterns^38^. The molecular and cellular events during ENS development in the embryonic stage may shed light on the pathogenesis of HSCR^39^. The *RET* gene, a key gene in type 2 multiple endocrine neoplasia^4^ and familial medullary thyroid carcinoma, has been considered a major gene in mediating susceptibility to HSCR. However, loss-of-function germ-line mutations in *RET* do not account for all of the HSCR cases. Therefore, genetic variations in other susceptible genes may be associated with HSCR. Neuregulins (NRGs) comprise a large family of EGF-like signaling molecules that are involved in cell-cell communication. NRGs transmit their signals to target cells by interacting with transmembrane tyrosine kinase receptors of the *ErbB* family. NRG-ErbB interactions activate intracellular signaling cascades, which induce cellular responses such as migration, proliferation, differentiation, and apoptosis. Some studies have demonstrated that the *ErbB* family plays an important role in the development of ENCCs^40^. Among the four family members (*NRG1, NRG2, NRG3* and *NRG4*), *NRG1* is expressed throughout the developing nervous system. *NRG3* shares the same receptor with *NRG1*, and these genes may work synergistically to affect the development of neuroblasts^41^.

In this case-controlled study of the Chinese population, we assayed eight polymorphisms at the *RET, NRG1*, and *NRG3* gene loci using whole blood specimens from 362 unrelated S-HSCR patients and 1,448 controls. We confirmed the association of the genetic variants in the *RET* proto-oncogene (rs2506030 and rs2435357) and *NRG1* (rs2439302 and rs7835688) with sporadic HSCR. Furthermore, this study provided insights into how these four SNPs interact with each other and jointly and/or synergistically increase the risk of HSCR development.

Intronic variation in rs2435357 appears to have a significant genetic association with HSCR in different ethnicities^35^,^36^,^42^, and this represents a functional site^43^. In our study, rs2435357 and rs2506030 were significantly associated with HSCR risk at the allele level and in different models (Table 2). Rs2435357 had a higher relative risk of HSCR in the allelic model (OR = 2.97). The prevalence of the rs2506030 susceptibility allele in the Chinese population is ~70%^38^, which is obviously higher than that in Africans (~8%) and Europeans (~40%). The different susceptibility allele frequencies may mean that the Chinese have a higher incidence of HSCR. The common *NRG1* genetic variant rs2439302 was initially reported by Gudmundsson *et al*.^33^. Here, we present its correlation with HSCR for the first time. *NRG1* encodes the signaling protein neuregulin 1 and is involved in the development of the nervous system and organs. It has been reported that each G allele confers a 40% decrease in *NRG1* expression compared to the C allele. Therefore, relative *NRG1* expression is significantly lower in GG carriers compared with CC carriers. It is reasonable to consider that rs2439302 may affect *NRG1* expression. This result may provide a basis for future studies on HSCR. In our study, logistic regression analysis revealed an increased risk associated with CG and GG carriers compared with CC carriers, and a significant association of rs2439302 and HSCR was found in dominant, recessive, additive and allelic models. Our results demonstrated that the rs7835688 SNP increased the risk for HSCR in our subjects. These associations do not agree with those in previous reports^38^,^44^, perhaps due to different races or anatomical types. Although some results support the hypothesis that the *NRG1* rs16879552 variant is a genetic risk factor for HSCR, the results in our study are inconsistent with these observations^26^,^32^. However, our findings correspond with those from studies in a Chinese population^37^ and Asian ancestry subjects^45^. The contradictory results may be due to the use of different samples from different population. We could not find any significant influence of variations in the three *NRG3* polymorphisms tested on the incidence of HSCR. In addition, the risk of HSCR was higher among patients with the heterozygote genotype and in the additive and dominant models (Table 2). However, none of these results reached significance in the present study. These findings suggest that *NRG3* polymorphisms do not confer a significant risk for HSCR. However, the results need to be confirmed with a larger patient population or a multicenter study.

The two variants rs2435357 and rs2506030 were identified as disrupting enhancers of *RET*, and they may play a similar role in affecting *RET* expression. The polymorphism rs2439302 at *NRG1* was shown to influence *NRG1* expression. However, with respect to the variations in these polymorphisms, there was a lesser influence on gene expression. Our western blotting and RT-qPCR results demonstrated that individuals carrying the risk genotypes had the lowest protein and mRNA expression levels of *RET* and *NRG1* in HSCR ganglionic colon tissues (Fig. 1). These results indicated that *RET* and *NRG1* protein and mRNA levels correlated with the different genotypes of the SNPs. It has been suggested that *NRG1* and *RET* play important roles in the pathogenesis of HSCR, and some data support the hypothesis that rs2506030 and rs2435357 variants disrupt *RET* enhancer activity^38^. However, further research is needed to elucidate how deficient expression results in aganglionosis.

In some previous studies, the cross-gene or cross-variant (common variants/rare variants) associations suggested joint effects or interactions with each other when certain variants coexist^10^,^23^. In the present study, we explored the interaction between *RET* and *NRG1* by analyzing combinations of SNPs involving these two genes. We found that these genetic interactions significantly enhanced the risk of HSCR in the study population. More importantly, compared with individuals whose genotype contained neither risk allele, the OR for the *NRG1* rs2439302 risk homozygote (GG) was increased by 25.69-fold in presence of the *RET* rs2506030 risk genotype (GG) and by 25.57-fold in the presence of the *RET* rs2435357 risk genotype (TT). *RET* and *NRG1* variants also showed genetic interactions with genotypes at three loci, revealing enhanced HSCR risk (OR = 56.53). Because of the high LD between the two variants rs7835688 and rs2439302 at the *NRG1* locus, the joint effects between rs7835688 and rs2435357 and/or rs2506030 were basically the same as those described above. Although functional assays supported genetic interactions between *NRG1* and *RET* that could be translated into functional interactions, the underlying molecular mechanism is still not well understood, and therefore, more sophisticated biological analyses are necessary to explain the complex interactions between these genes.

Despite the sufficient power of the comprehensive analysis in our study, several limitations should be acknowledged. First, the sample size of our case-controlled study was relatively small. Second, HSCR is a complex disease, affected by interactions between genetic and environmental factors. The unavailability of adequate clinical information limited our ability to further investigate the interactions.

In conclusion, our study showed for the first time that the functional common genetic variant rs2439302 in *NRG1* might confer altered risk to HSCR in the Chinese population and provided genotype-phenotype correlations regarding *RET* and *NRG1* expression levels in HSCR. We also demonstrated the existence of cumulative genetic risk for HSCR between *RET* and *NRG1* polymorphisms in the Chinese population. These results may provide a basis for further biological and/or molecular studies of HSCR.

## Methods

### Ethics statement

This research project has been approved by the Ethical Committee of the Medical Association of Tongji Medical College, Huazhong University of Science and Technology. Informed consent was obtained from all participants or legal guardians.

### Subjects

We selected a total of 362 patients with sporadic S-HSCR from Wuhan Union Hospital and 1,448 healthy individuals as controls who were randomly recruited from the general population in the Wuhan region of Hubei Province, China. The diagnosis of HSCR was based on histological examination of either biopsy tissues or pathology specimens obtained during operations. A 2 ml sample of peripheral venous blood was collected from the patients and the controls, and the blood specimens were mixed with the anti-coagulant ethylene diamine tetraacetic acid (EDTA). Then, genomic DNA was extracted from the white blood cells using a QIAamp DNA Blood Midi Kit (Qiagen, Hilden, Germany) according to the manufacturer’s protocols. DNA concentrations were determined with a UV spectrophotometer, and the samples were stored at −80 °C until use. Full-thickness ganglionic colon tissues were obtained from patients who were pathologically confirmed to have HSCR and were stored at −80 °C.

### Genotyping

In our study, we selected eight SNPs to assess the risk association with HSCR: rs2435357 and rs2506030 at RET; rs2439302, rs7835688 and rs16879552 at *NRG1*; and rs10748842, rs10883866 and rs6584400 at *NRG3*. The genetic polymorphisms were genotyped using TaqMan SNP Genotyping Assays (Applied Biosystems, Foster City, CA) on a 7900HT Fast Real-Time PCR System (Applied Biosystems, Foster City, CA). The assay IDs are as follows: rs2506030 (C_26742714_10), rs2435357 (C_16017524_10), rs2439302 (C_16238367_10), rs7835688 (C_32689004_10), rs16879552 (C_32689001_10), rs10748842 (C_1266043_10), rs10883866 (C_31751237_10), and rs6584400 (C_30519098_10). The TaqMan probes were VIC and FAM dye-labeled. Available oligonucleotide sequences are shown in Table 5. Genotype data were analyzed in a blinded manner regading the case-controlled status. For quality control, we randomly selected 5% duplicated samples for sequencing to assess the reproducibility, with a concordance rate of 100%. All the experimental methods were conducted in accordance with the approved guidelines.

### RNA isolation and RT-qPCR

Total RNA was extracted from frozen HSCR ganglionic colon tissues using TRIzol Reagent (Invitrogen, Life Technologies Inc., Carlsbad, CA, USA) according to the manufacturer’s instructions. For mRNA detection, 500 ng of total RNA was reverse transcribed to cDNA using a reverse transcription kit (Takara, Tokyo, Japan). The expression levels of the *NRG1* and *RET* genes were measured by qPCR using a 7900HT Fast Real-Time PCR System (Applied Biosystems, Foster City, CA) according to the manufacturer’s protocols. The *NRG1* and *RET* mRNA expression levels were normalized to *GAPDH* expression, and relative gene expression was calculated using the 2^−ΔΔCt^ method. Forward (F) and reverse (R) primer sequences for each gene were as follows: *NRG1*, (F) 5′-ATGTGTCTTCAGAGTCTCCCAT-3′ and (R) 5′-TGGACGTACTGTAGAAGCTGG-3′; *RET*, (F) 5′-CTGCCAAGTCCCGATG-3′ and (R) 5′-TGGAGTACGCCAAATACG-3′; and *GAPDH*, (F) 5′-CAATGACCCCTTCATTGACC-3′ and (R) 5′-GACAAGCTTCCCGTTCTCAG-3′.

### Western blotting

Total protein was extracted from HSCR ganglionic colon tissues with a radio-immunoprecipitation assay (RIPA) buffer containing protease inhibitors (cOmplete, ULTRA, 132Mini, EDTA-free, EASY pack; Roche, Basel, Switzerland). Protein samples were boiled at 95 °C for 5 min, cooled at room temperature for 5 min and then centrifuged. Protein concentrations were determined using the BCA (bicinchoninic acid) method. Equal amounts of protein (50 μg) were separated by 12.5% sodium dodecyl sulfate polyacrylamide gel electrophoresis (SDS-PAGE) and then transferred to a polyvinylidene fluoride (PVDF) membrane (Roche, Basel, Switzerland). Membranes were blocked using 5% skimmed milk and incubated with the appropriate diluted antibodies (anti-*NRG1*, 1:1000, #ab27303; anti-*RET*, 1:1000, #ab134100; Abcam, Cambridge, UK). Membranes were subsequently incubated with a 1:4,000 dilution of a horseradish peroxidase-conjugated secondary antibodies (Invitrogen, Logan, UT, USA) for 1 h, followed by 3 washes with TBST for 15 min. Membranes were then processed using enhanced chemiluminescence (ECL) (Bio-Rad, Hercules, CA, USA) and were exposed to film (Canon, Tokyo, Japan). The experiments were repeated 3 times. The protein expression levels of *NRG1* and *RET* were normalized to those of *GAPDH*. All the experimental procedures were conducted according to the manufacturer’s recommended instructions.

### Statistical analysis

The statistical analyses were performed with SPSS version 21.0 (SPSS Inc., Chicago, IL, USA). The Hardy–Weinberg equilibrium of the three SNPs was assessed with a goodness of fit Chi-squared test in the control subjects. To analyze the frequency of alleles and genotypes in all samples, the Chi-square test was used. The ORs and 95% CIs were determined for the association between disease status and genotype using a Chi-squared test, and two-tailed P-values less than 0.05 were considered significant. Dominant, recessive and additive models for the three SNPs in association with HSCR were analyzed to avoid the assumptions of genetic models. The wild type was considered as a reference. The genetic comparisons included a homozygous model, heterozygous model, dominant model and recessive model. The combined effects of *NRG1* (rs2439302) and *RET* SNPs (rs2506030 and rs2435357) on HSCR risk were assessed. ORs and the frequency of the SNP alleles were calculated for disease markers. We used Haploview 4.2 for tests of LD determined by the r^2^ value. For SNP-SNP interactions, we used a multiple logistic regression model to estimate the multiplicative interaction effect of the SNPs. All data are shown in Fig. 1 as the mean ± standard error of the mean (SEM).

## Additional Information

**How to cite this article:** Yang, D. *et al*. Effects of RET, *NRG1* and *NRG3* Polymorphisms in a Chinese Population with Hirschsprung Disease. *Sci. Rep.* **7**, 43222; doi: 10.1038/srep43222 (2017).

**Publisher's note:** Springer Nature remains neutral with regard to jurisdictional claims in published maps and institutional affiliations.

## Supplementary Material

Supplementary Information

## Figures and Tables

**Table 1:** Clinical characteristics of the subjects.

	Case (No.)	%	Control (No.)	%	*P*
Total	362		1448		
Gender					1.00
Male	275	75.97	1100	75.97	
Female	87	24.03	348	24.03	

**Table 2:** Allele and genotype distributions among HSCR patients and normal controls and their association with HSCR.

Genotype	Case		Control		OR*(95%CI)	*P*
	No.	(%)	No.	(%)		
**rs2435357**						
CC	27	7.5	317	21.9	1.00	
CT	126	34.8	802	55.4	1.85 (1.19–2.85)	5.86E-03
TT	209	57.7	329	22.7	7.46 (4.85–11.46)	4.74E-20
T/C					2.97 (2.47–3.57)	5.15E-33
Additive model					3.25 (2.67–3.96)	1.32E-31
Recessive model					4.63 (3.63–5.90)	2.35E-35
Dominant model					3.48 (2.31–5.25)	2.89E-09
T allele frequency	0.751		0.504			
**rs2506030**						
AA	13	3.8	143	9.9	1.00	
AG	111	32.2	550	38.0	2.22 (1.21–4.06)	0.01
GG	221	64.1	755	52.1	3.22 (1.79–5.79)	9.50E-05
G/A					1.64 (1.34–2.00)	1.72E-06
Additive model					1.60 (1.31–1.95)	4.47E-06
Recessive model					1.64 (1.28–2.09)	7.13E-05
Dominant model					2.80 (1.57–5.00)	1.00E-03
G allele frequency	0.801		0.711			
**rs2439302**						
CC	185	51.8	950	65.6	1.00	
CG	133	37.3	458	31.6	1.49 (1.16–1.91)	0.002
GG	39	10.9	40	2.8	5.01 (3.14–8.00)	1.57E-11
G/C					1.84 (1.53–2.21)	9.36E-11
Additive model					1.85 (1.53–2.23)	2.15E-10
Recessive model					4.32 (2.73–6.82)	3.70E-10
Dominant model					1.77 (1.40–2.24)	1.64E-06
G allele frequency	0.296		0.186			
**rs7835688**						
GG	193	53.3	989	68.3	1.00	
GC	120	33.1	389	26.9	1.58 (1.22–2.04)	4.62E-04
CC	49	13.6	70	4.8	3.59 (2.41–5.33)	2.66E-10
C/G					1.93 (1.60–2.32)	1.88E-12
Additive model					1.78 (1.50–2.12)	9.71E-11
Recessive model					3.08 (2.10–4.53)	1.02E-08
Dominant model					1.89 (1.49–2.39)	1.09E-07
C allele frequency	0.301		0.183			

CI = confidence interval. ^*^ORs and 95% CIs were calculated by unconditional logistic regression.

**Table 3:** Joint effect tests of RET rs2435357 or rs2506030 and NRG1 rs2439302 polymorphisms in HSCR.

Gene-gene							
SNP-SNP interaction	Combination		Frequency No. (%)		Cases	OR* (95% CI)	*P*
RET	NRG1	Genotype	Genotype	Control			
rs2506030	rs2439302	AA	CC	111 (7.67)	11 (3.19)	Reference	
		—	CG	28 (1.93)	1 (0.29)	0.36 (0.045–2.91)	3.38E-01
		—	GG	4 (0.27)	1 (0.29)	2.52 (0.26–24.60)	4.26E-01
		AG	CC	345 (23.83)	65 (18.84)	1.90 (0.97–3.73)	6.17E-02
		—	CG	180 (12.43)	36 (10.43)	2.02 (0.99–4.13)	5.44E-02
		—	GG	25 (17.27)	10 (2.90)	4.04 (1.55–10.54)	4.39E-03
		GG	CC	494 (34.11)	103 (29.86)	2.10 (1.09–4.05)	2.60E-02
		—	CG	250 (17.27)	90 (26.09)	3.63 (1.87–7.06)	1.43E-04
		—	GG	11 (0.76)	28 (8.12)	25.69 (10.11–65.29)	9.12E-12
rs2435357	rs2439302	CC	CC	225 (15.54)	22 (6.16)	Reference	
		—	CG	79 (5.46)	3 (0.84)	0.39 (0.11–1.33)	1.33E-01
		—	GG	13 (0.97)	2 (0.56)	1.57 (0.33–7.43)	5.67E-01
		CT	CC	515 (35.57)	75 (21.00)	1.49 (0.90–2.46)	1.19E-01
		—	CG	270 (18.64)	39 (10.92)	1.48 (0.85–2.57)	1.66E-01
		—	GG	17 (1.17)	12 (3.36)	7.22 (3.06–17.04)	6.46E-06
		TT	CC	210 (14.50)	88 (24.65)	4.29 (2.59–7.09)	1.48E-08
		—	CG	109 (7.53)	91 (25.49)	8.54 (5.08–14.34)	5.40E-16
		—	GG	10 (0.69)	25 (7.00)	25.57 (10.88–60.07)	1.02E-13

^*^ORs and 95% CIs were calculated by unconditional logistic regression. CI = confidence interval.

**Table 4:** The joint effects of SNP-SNP interactions and HSCR risk.

SNP-SNP Combination			Frequency No. (%)		OR* (95% CI)	*P*
rs2506030 Genotype	rs2439302 Genotype	rs2435357 Genotype	Control	Cases		
AA	CC	CC	53 (3.66)	5 (1.45)	Reference	
—	—	CT	35 (2.42)	3 (0.87)	0.91 (0.20–4.05)	8.99E-01
—	—	TT	23 (1.59)	3 (0.87)	1.38 (0.31–6.28)	6.75E-01
—	CG	CC	12 (0.83)	0 (0.00)	N.S.	N.S.
—	—	CT	10 (0.69)	1 (0.29)	1.06 (0.11–10.07)	9.56E-01
—	—	TT	6 (0.41)	0 (0.00)	N.S.	N.S.
—	GG	CC	1 (0.07)	0 (0.00)	N.S.	N.S.
—	—	CT	2 (0.14)	0 (0.00)	N.S.	N.S.
—	—	TT	1 (0.07)	1 (0.29)	10.60 (0.57–196.45)	1.13E-01
AG	CC	CC	58 (4.00)	4 (1.16)	0.73 (0.19–2.87)	6.53E-01
—	—	CT	222 (15.33)	24 (6.96)	1.15 (0.42–3.14)	7.91E-01
—	—	TT	65 (4.49)	37 (10.72)	6.03 (2.22–16.43)	4.37E-04
—	CG	CC	47 (3.25)	1 (0.29)	0.23 (0.025–2.00)	1.81E-01
—	—	CT	71 (4.90)	9 (2.61)	1.79 (0.60–5.39)	3.00E-01
—	—	TT	62 (4.28)	26 (7.54)	3.93 (1.40–11.06)	9.47E-03
—	GG	CC	7 (0.48)	1 (0.29)	1.51 (0.15–14.91)	7.23E-01
—	—	CT	8 (0.55)	3 (0.87)	5.29 (1.17–24.00)	3.05E-02
—	—	TT	10 (0.69)	6 (1.74)	5.30 (1.29–21.75)	2.06E-02
GG	CC	CC	82 (5.66)	8 (2.32)	1.03 (0.32–3.33)	9.56E-01
—	—	CT	319 (22.03)	37 (10.72)	1.23 (0.46–3.27)	6.79E-01
—	—	TT	93 (6.42)	62 (17.97)	6.61 (2.50–17.51)	1.44E-04
—	CG	CC	53 (0.37)	2 (0.58)	0.40 (0.07–2.15)	2.86E-01
—	—	CT	131 (9.05)	34 (9.86)	2.75 (1.02–7.41)	4.54E-02
—	—	TT	66 (4.56)	54 (15.65)	8.67 (3.24–23.22)	7.03E-06
—	GG	CC	4 (0.28)	3 (0.87)	7.95 (1.37–46.00)	2.06E-02
—	—	CT	4 (0.28)	9 (2.61)	23.85 (5.36–106.10)	3.12E-05
—	—	TT	3 (0.21)	16 (4.64)	56.53 (12.16–262.83)	4.50E-07

^*^ORs and 95% CIs were calculated by unconditional logistic regression. N.S. = Not significant.

**Table 5:** Oligonucleotide sequences of PCR amplification primers and TaqMan probes for genotyping the eight SNPs studied.

SNP_ID	Position	Sequence name	Sequence	Genotype
rs2435357	RET	rs2435357_F	5′-GAGTGCATGGGGACAGTT-3'	C/T
		rs2435357_R	5′-GGAAACTGCCAATTAGGTTAT-3'	
		rs2435357_VIC	5′-TGGATGACCATGTAAGGG-3'	
		rs2435357_FAM	5′-TGGATGACCGTGTAAGGG-3'	
rs2506030	RET	rs2506030_F	5′-GCGACTGAATGAAGCACTCTGA-3'	A/G
		rs2506030_R	5′-ATGTGGCTCTGCTGGTTTCTG-3'	
		rs2506030_VIC	5′-ACACCAATGACCTGTT-3'	
		rs2506030_FAM	5′-ACACCAATGACCTATT-3'	
rs2439302	NRG1	rs2439302_F	5′-CATAGGAGAGTTAGGTGGCAAAGC-3'	C/G
		rs2439302_R	5′-CAAGAATGGCCTAACACAATGTG-3'	
		rs2439302_VIC	5′-TAGTGTAAACTGTATGAAAC-3'	
		rs2439302_FAM	5′-TAGTGTAAACTCTATGAAAC-3'	
rs7835688	NRG1	rs7835688_F	5′-TCAATGTTCTGTTAGCATTCT-3'	G/C
		rs7835688_R	5′-TGTTTTCATCTATCCTAACAGAACA-3'	
		rs7835688_VIC	5′-ACAAGTTAAATTCGATTT-3'	
		rs7835688_FAM	5′-AACAAGTTAAATTGGATT-3'	
rs16879552	NRG1	rs16879552_F	5′-AGTTGATGCTTAAACCTCACTTTATT-3'	T/C
		rs16879552_R	5′-AGGTTGGTGCACACTTTTGTTTT-3'	
		rs16879552_VIC	5′-TTTTGGGACTTGTATAAATT-3'	
		rs16879552_FAM	5′-TTTGGGACTCGTATAAATTG-3'	
rs10748842	NRG3	rs10748842_F	5′-TGATGCTCATCTTGCATTGTGA-3'	T/C
		rs10748842_R	5′-TGTATTGGAGGAAACAGCATTTCA-3'	
		rs10748842_VIC	5′-CTCTGAGTAATTTATG-3'	
		rs10748842_FAM	5′-AGGCTCTGAGTAACTT-3'	
rs10883866	NRG3	rs10883866_F	5′-CCACAGCACGGAACA-3'	C/G
		rs10883866_R	5′-TAGCGGAGGAGGAAGA-3'	
		rs10883866_VIC	5′-ATAATCAGTCTTGGAAGGGGCT-3'	
		rs10883866_FAM	5′-ATAATCAGTGTTGGAAGGGGCT-3'	
rs6584400	NRG3	rs6584400_F	5′-AAGGCTTCGGCTTCT-3'	G/A
		rs6584400_R	5′-CAACTCAACACCACCAAT-3'	
		rs6584400_VIC	5′-AGCATGCCTGATTCCTCTTCCA-3'	
		rs6584400_FAM	5′-AAGCATGCCTAATTCCTCTTCC-3'	
