# Supplementary material for: Effects of RET, NRG1 and NRG3 Polymorphisms in a Chinese Population with Hirschsprung Disease
Source: Sci Rep. 2017 Mar 3;7:43222. doi: 10.1038/srep43222 (PMC5335705; doi:10.1038/srep43222)

Effects of *RET*, *NRG1* and *NRG3* Polymorphisms in a Chinese Population with Hirschsprung Disease

Dehua Yang[1,+], Jun Yang[2,+], Shuai Li[1,+], Meng Jiang[1],Guoqing Cao[1], Li Yang[1], Xi Zhang[1], Ying Zhou[1], Kang Li[1], Shao-tao Tang[1,*]

Author Affiliations

[1]Department of Pediatric Surgery, Union Hospital, Tongji Medical College, Huazhong University of Science and Technology, Wuhan 430022, China

[2]Department of Pediatric Surgery, Wuhan Children's Hospital (Wuhan Maternal and Child Healthcare Hospital), Tongji Medical College, Huazhong University of Science and Technology, Wuhan 430015, China

[*]Correspondence: Dr. Shao-tao Tang, Department of Pediatric Surgery, Union Hospital, Tongji Medical College, Huazhong University of Science and Technology, Wuhan 430022, China. Tel.: +8613720313268, E-mail: tshaotao83@126.com

[+]These authors contributed equally to this work.

Supplementary Table S1. Genotype frequencies of *NRG1* (rs16879552) and *NRG3* (rs10748842, rs10883866 and rs6584400) polymorphisms among patients and normal controls and their associations with HSCR in Chinese population. CI=confidence interval.

Genotype	Case		Control		OR*(95%CI)	*P*
	No.	(%)	No.	(%)		
**rs16879552**						
TT	138	38.2	565	39.0	1.00	
TC	128	35.3	560	38.7	0.94(0.72-1.22)	6.27E-01
CC	96	25.6	323	22.3	1.22(0.91-1.63)	1.91E-01
C/T					1.11(0.94-1.31)	2.13E-01
Additive model					1.09(0.94-1.26)	2.60E-01
Recessive model					1.26(0.97-1.64)	8.96E-02
Dominant model					1.04(0.82-1.32)	7.54E-01
C allele frequency	0.442		0.416			
**rs10748842**						
TT	298	83.2	1237	85.4	1.00	
TC	58	16.2	202	14.0	1.20(0.87-1.64)	2.79E-01
CC	2	0.6	9	0.6	0.92(0.20-4.29)	9.17E-01
C/T					1.15(0.86-1.55)	3.43E-01
Additive model					1.15(0.86-1.55)	3.43E-01
Recessive model					0.90(0.19-4.18)	8.91E-01
Dominant model					1.18(0.86-1.62)	3.00E-01
C allele frequency	0.087		0.076			
**rs10883866**						
CC	320	88.4	1304	90.1	1.00	
CG	32	8.8	102	7.0	1.28(0.84-1.94)	2.47E-01
GG	10	2.8	42	2.9	0.97(0.48-1.96)	9.33E-01
G/C					1.13(0.82-1.55)	4.61E-01
Additive model					1.09(0.83-1.43)	5.33E-01
Recessive model					0.95(0.47-1.91)	8.88E-01
Dominant model					1.19(0.83-1.71)	3.53E-01
G allele frequency	0.072		0.064			
**rs6584400**						
GG	207	57.2	872	60.2	1.00	
GA	137	37.8	521	36.0	1.11(0.87-1.41)	4.07E-01
AA	18	5.0	55	3.8	1.38(0.79-2.40)	2.56E-01
A/G					1.13(0.93-1.37)	2.23E-01
Additive model					1.14(0.93-1.38)	2.11E-01
Recessive model					1.32(0.77-2.27)	3.11E-01

Dominant model			1.13(0.93-1.38)	2.81E-01
A allele frequency	0.239	0.218		

Supplementary Table S2. Joint effect tests of RET rs2435357-TT and/or rs2506030-GG and NRG1 rs7835688-CC in HSCR. *ORs and 95% CIs were calculated by unconditional logistic regression. CI=confidence interval.

**SNP-SNP Combination**			**Frequence N, (%)**			
**rs7835688 Genotype**	**rs2506030 Genotype**	**rs2435357 Genotype**	**Control**	**Patients**	**OR ratio (95% CI)**	***P*-value**
CC	GG	-	14(0.97)	32(9.28)	24.13(9.54-61.05)	1.80E-11
CC	-	TT	15(0.97)	18(8.09)	24.10(9.82-59.13)	3.68E-12
CC	GG	TT	3(0.21)	14(4.06)	54.83(10.94-274.74)	1.12E-06

Supplementary Figure S3. Full-size blots of *RET*, *NRG1* and *GAPDH* expressed in three genotypes of rs2435357, rs2506030 and rs2439302.

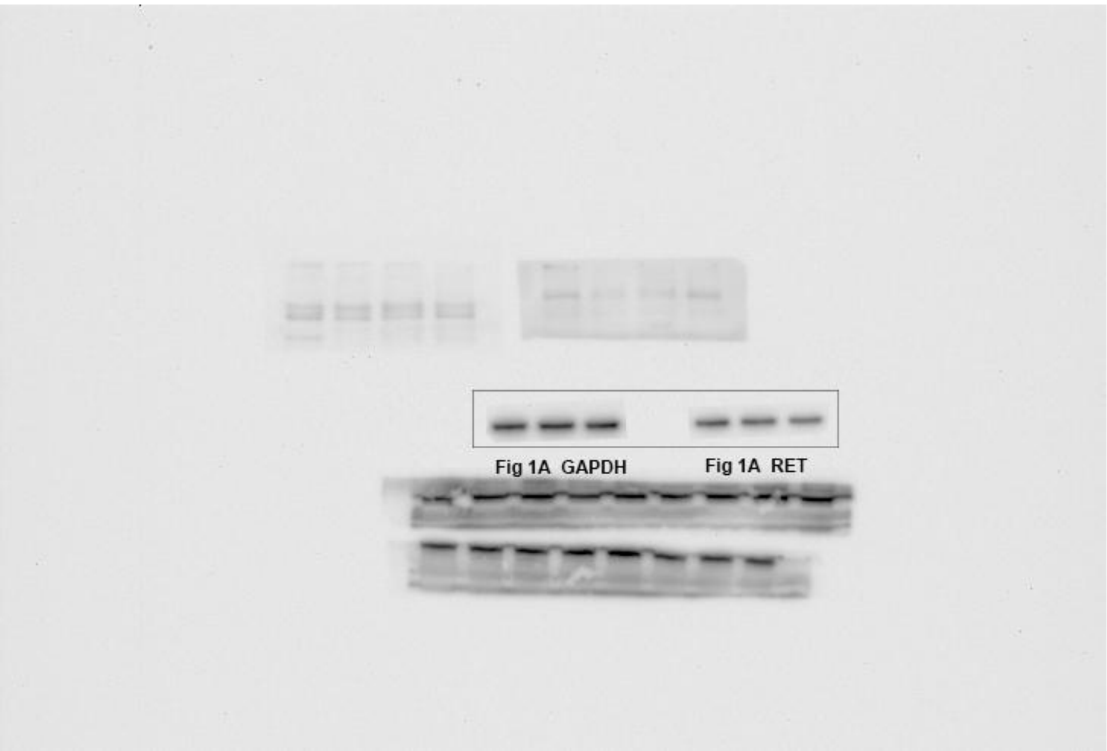

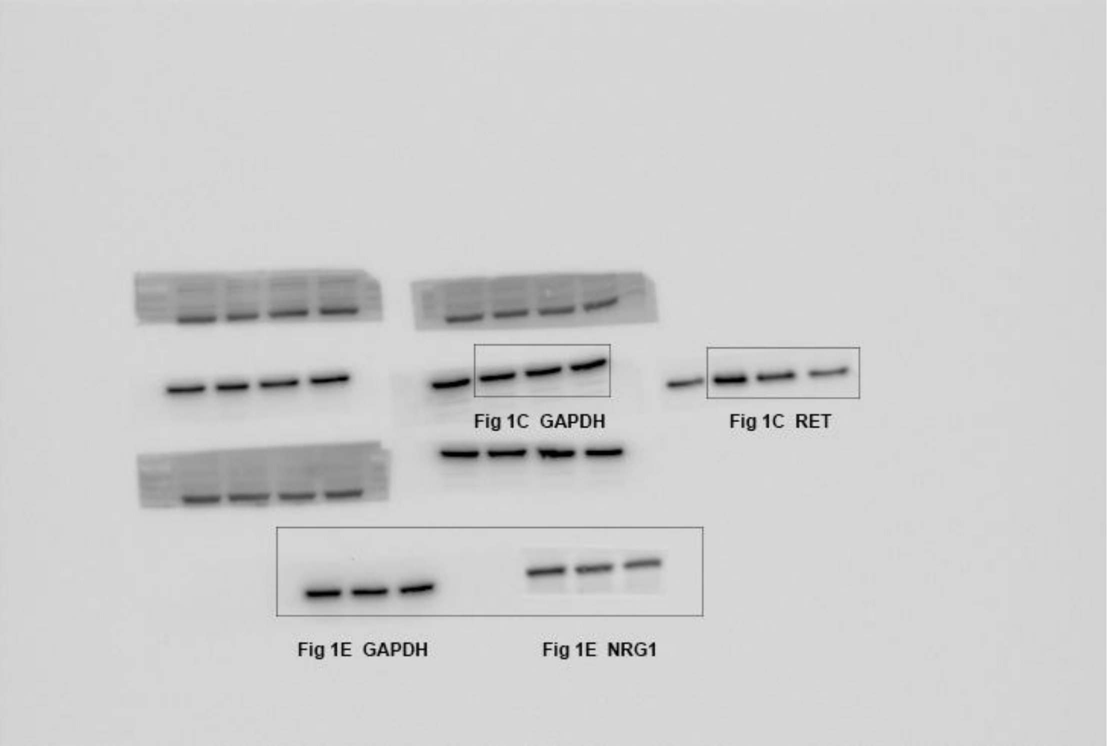

Supplement: Supplementary Information [file srep43222-s1.pdf]
